# ATP Induced Brain-Derived Neurotrophic Factor Expression and Release from Osteoarthritis Synovial Fibroblasts Is Mediated by Purinergic Receptor P2X4

**DOI:** 10.1371/journal.pone.0036693

**Published:** 2012-05-25

**Authors:** Kerstin Klein, André Aeschlimann, Suzana Jordan, Renate Gay, Steffen Gay, Haiko Sprott

**Affiliations:** 1 Center of Experimental Rheumatology, Division of Rheumatology and Institute of Physical Medicine, University Hospital Zurich, Zurich, Switzerland; 2 Zurich Center of Integrative Human Physiology (ZIHP), Zurich, Switzerland; 3 RehaClinic, Bad Zurzach, Switzerland; University of Western Ontario, Canada

## Abstract

Brain-derived neurotrophic factor (BDNF), a neuromodulator involved in nociceptive hypersensitivity in the central nervous system, is also expressed in synoviocytes of osteoarthritis (OA) and rheumatoid arthritis (RA) patients. We investigated the role of P2 purinoreceptors in the induction of BDNF expression in synovial fibroblasts (SF) of OA and RA patients. Cultured SF from patients with symptomatic knee OA and RA were stimulated with purinoreceptor agonists ATP, ADP, or UTP. The expression of BDNF mRNA was measured by quantitative TaqMan PCR. BDNF release into cell culture supernatants was monitored by ELISA. P2X4 expression in synovial tissue was detected by immunohistochemistry. Endogenous P2X4 expression was decreased by siRNA transfection before ATP stimulation. Kinase pathways were blocked before ATP stimulation. BDNF mRNA expression levels in OASF were increased 2 h and 5 h after ATP stimulation. Mean BDNF levels in cell culture supernatants of unstimulated OASF and RASF were 19 (±9) and 67 (±49) pg/ml, respectively. BDNF levels in SF supernatants were only elevated 5 h after ATP stimulation. BDNF mRNA expression in OASF was induced both by P2X receptor agonists ATP and ADP, but not by UTP, an agonist of P2Y purinergic receptors. The ATP-induced BDNF mRNA expression in OASF was decreased by siRNA-mediated reduction of endogenous P2X4 levels compared to scrambled controls. Inhibition of p38, but not p44/42 signalling reduced the ATP-mediated BDNF mRNA induction. Here we show a functional role of the purinergic receptor P2X4 and p38 kinase in the ATP-induced expression and release of the neurotrophin BDNF in SF.

## Introduction

Osteoarthritis (OA) and rheumatoid arthritis (RA), the most frequent joint diseases, are typically accompanied by chronic pain. Several studies demonstrated that the concordance between joint pain and radiological abnormality is poor [1]. There is mounting evidence indicating that compounds which act as mediators of pain in the nervous system also play a functional role in the synovial tissue. Synovial fibroblasts (SF) were shown to release an array of cytokines and growth factors that act on diverse cells including neurons. Therefore, SF have the capacity to modulate inflammation and promote matrix degradation and to play a central role in the development of joint pain [2]. In many studies, OASF are compared with RASF, due to the lack of normal SF from healthy individuals [3].

Brain-derived neurotrophic factor (BDNF) is known as a crucial neuromodulator involved in nociceptive hypersensitivity in the central nervous system [4] and BDNF levels are modified in some persistent pain states as well as in inflammation [5], [6], [7], [8]. BDNF exerts its action via binding to the high affinity receptor tropomyosine receptor kinase B (TrkB) or the low affinity receptor p75^NTR^, which is capable of binding all neurotrophins [9]. The function of BDNF in the central nervous system has been studied in detail [10]. Recently, a role of neurotrophins as important factors in arthritis has been discussed [11]. High BDNF mRNA expression levels as well as expression of the receptors TrkB and p75^NTR^ were detected in synovial fluid cells of OA, RA and spondyloarthritis patients [12]. Moreover, a positive immunostaining for BDNF, TrkB and p75^NTR^ in synovial tissue sections of OA and RA patients was reported [13]. TrkB staining was detected in nerve fibres in synovial tissue sections of knee OA and RA patients [13], [14]. In addition, TrkB expression was shown in articular chondrocytes and inflammatory infiltrates in knee joints of local injection-induced mice [15]. An increased BDNF immunostaining in SF and macrophages in synovial tissue of OA and RA patients compared to healthy controls has been reported previously [16], but the regulation of BDNF expression in SF is still unknown.

Extracellular ATP levels are increased in inflamed or damaged tissues and the nucleotide is found in the synovial fluid of arthritis patients [17]. ATP signalling is mediated by P2 purinoreceptors that are divided into two families, ionotropic receptors P2X and metabotropic receptors P2Y. P2X receptors (P2X1–P2X7) contain intrinsic pores that open by binding ATP, and P2Y receptors (P2Y1, 2, 4, 6, 11, 12, 13, 14) are activated by nucleotides and couple to intracellular second-messenger systems through heterotrimeric G-proteins. Recently, the P2X4 receptor-mediated release of BDNF from rat microglia was shown to be dependent on calcium and p38 mitogen-activated protein kinase (MAPK) [18]. We aimed to investigate whether SF are capable of secreting BDNF and to identify the underlying mechanism. Here, we report a functional role of the purinergic receptor P2X4 in the ATP-induced expression and release of the neurotrophin BDNF in SF.

## Results

### ATP Induces the Expression of BDNF in SF of Arthritic Patients

**Figure 1:**
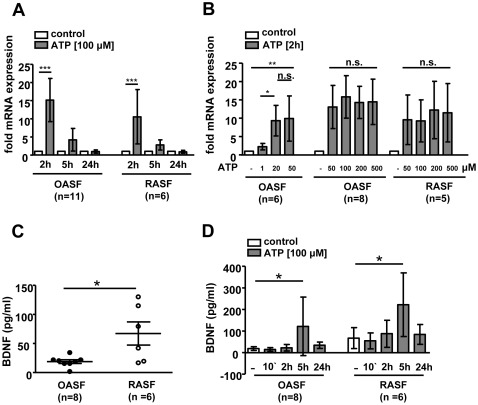
ATP-induced BDNF expression in SF. OASF (n = 11) and RASF (n = 6) were stimulated with ATP (50, 100, 200, 500 µM) for 10 minutes, 2 h, 5 h and 24 h. A. BDNF mRNA levels were induced 2 h after ATP stimulation. B. Influence of different ATP concentrations on the induction levels of BDNF mRNA after 2 h ATP stimulation. C. BDNF protein levels in cell culture supernatants of unstimulated OASF (n = 8) and RASF (n = 6) were measured by ELISA. D. BDNF protein levels in cell culture supernatants of OASF and RASF were elevated 5 h after ATP stimulation. Data are shown as means ± standard deviations. N.s., not significant; *, p<0.05, ***, p<0.001.

Time course experiments revealed an increased BDNF mRNA expression in OASF 2 h (15.2±6.0 fold, n = 11, p<0.001) and 5 h (4.2±3.1 fold, n.s.) after ATP stimulation compared to unstimulated cells (Fig. 1A). After 24 h ATP stimulation, BDNF mRNA levels returned to control levels. In RASF, BDNF mRNA levels were also elevated 2 h (10.8±6.9 fold, n = 6, p<0.001) and 5 h (2.6±1.4 fold, n.s.) after ATP stimulation (Fig. 1A). This effect was not dose-dependent at high µM ATP concentrations (Fig. 1B), since the reduction of ATP to a concentration of 50 µM or a further increase to 200 µM and 500 µM ATP did not affect BDNF mRNA levels in 2 h stimulated OASF (n = 8) and RASF (n = 5). Whereas 1 µM ATP only mildly stimulated BDNF mRNA expression, a concentration of 20 µM ATP was sufficient to completely activate BDNF mRNA expression in OASF (n = 6). In order to validate the effect of elevated ATP concentrations on BDNF protein levels, BDNF was measured in cell culture supernatants of stimulated and unstimulated OASF (n = 11) and RASF (n = 6). Mean BDNF levels in cell culture supernatants of unstimulated OASF (n = 8) and RASF (n = 6) were 19 (±9) and 67 (±49) pg/ml (p<0.05), respectively (Fig. 1C). Outliers from three OA patients were excluded from this analysis since these patients showed 14-fold increased BDNF levels (266±36 pg/ml) compared to all other OASF. Interestingly, these three patients were the only ones that have received centrally acting drugs. BDNF levels in OASF supernatants (Fig. 1D) were increased (122±136 pg/ml, p<0.05) 5 h after ATP stimulation. Similar results were obtained for RASF (222±148 pg/ml, p<0.05) after 5 h ATP stimulation.

### ATP and ADP, but not UTP Induce BDNF Expression

**Figure 2:**
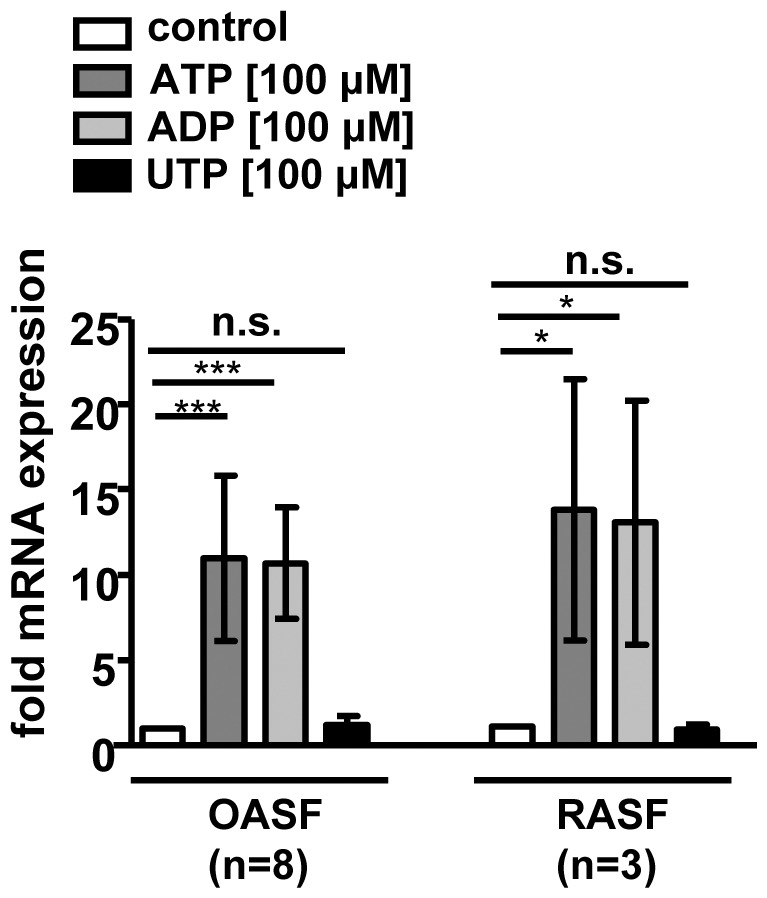
Purinergic receptor P2X, but not P2Y agonists induce BDNF expression. OASF (n = 8) and RASF (n = 3) were stimulated for 2 h with 100 µM ATP, ADP and UTP, respectively. BDNF mRNA expression was increased by ATP and ADP, but not by UTP. Data are shown as means ± standard deviations. N.s., not significant; *, p<0.05, ***, p<0.001.

Expression of several P2 purinoreceptors in OA and RA synoviocytes was, at least on the mRNA level, reported previously [19], [20]. In order to narrow the number of possibly involved receptors down that mediate the induction of BDNF, OASF (n = 8) and RASF (n = 3) were stimulated with purinoreceptor agonists ATP, ADP or UTP for 2 h. BDNF mRNA levels were induced by P2X receptor agonists ATP (OASF: 11.0±4.3 fold, p<0.001; RASF: 13.4±6.3 fold, p<0.05) and ADP (OASF: 10.5±2.9 fold, p<0.001; RASF: 12.9±5.8 fold, p<0.05), but not UTP (OASF: 1.2±0.5 fold, n.s.; RASF: 0.8±0.3 fold, n.s.), a P2Y receptor agonist (Fig. 2). These results highly suggested a role for the purinoreceptor P2X4 in the ATP-induced regulation of BDNF expression. The probability for the involvement of other P2X receptors was low, since they preferentially respond to either much lower ATP concentrations (P2X1,3) or high ATP concentrations (P2X7), respectively [21].

### The P2X4 Receptor is Expressed in SF

**Figure 3:**
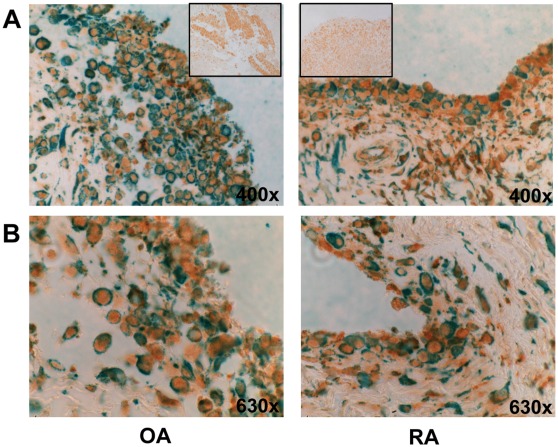
Immunohistochemistry. Representative photomicrographs showing immunohistochemical double staining of OA and RA synovial tissues with P2X4 (brown) and fibroblast marker (green). **A**. original magnification 400×, box shows the Isotype control, magnification 200×. **B**. original magnification 630×.

Since the expression of P2X4 in SF was so far only verified on mRNA levels [20], we investigated the expression of this receptor (brown) by immunohistochemistry (Fig. 3) and double stained with a fibroblast marker (green). Our double staining of OA and RA synovial tissue (n = 4) revealed that the P2X4 receptor is present in SF but also in other non-fibroblast cell types.

### The Receptor P2X4 is Involved in the ATP-induced Expression of BDNF

**Figure 4:**
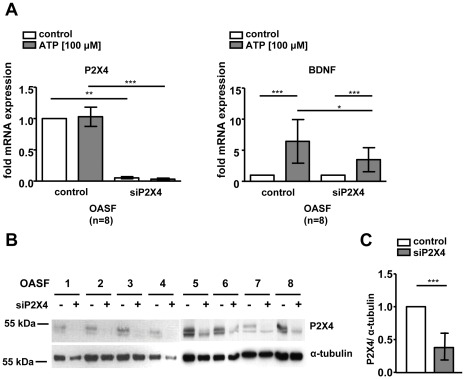
Purinergic receptor P2X4 siRNA transfection in OASF (n = 8). Endogenous P2X4 expression in OASF was reduced by siRNA transfection before ATP (100 µM) stimulation for 2 h. **A**. P2X4 mRNA expression was reduced compared to scrambled controls. The ATP-induced BDNF mRNA expression in P2X4 siRNA transfected cells was reduced compared to scrambled controls. **B**. P2X4 protein levels were reduced by P2X4 siRNA transfection compared to scrambled controls. α-tubulin levels in P2X4 siRNA transfected cells were slightly decreased compared to controls due to experimental conditions. **C**. Densitometric analysis of Western blot results. Data are shown as means ± standard deviations. N.s., not significant; *, p<0.05, ***, p<0.001.

In order to investigate the potential role of P2X4 in the ATP-mediated induction of BDNF, we reduced endogenous P2X4 levels in OASF (n = 8) using siRNA transfections and repeated the ATP stimulation experiments. The ATP-induced BDNF mRNA expression was decreased (control: 6.4±3.5 fold, siP2X4: 3.5±1.9 fold, p<0.05) by siRNA-mediated reduction of endogenous P2X4 levels in OASF compared to scrambled controls (Fig. 4A). The reduction of P2X4 mRNA and protein expression levels were verified by Real-time PCR (Fig. 4A) as well as Western blotting (Fig. 4B) followed by densitometric analysis of band intensities. Endogenous P2X4 levels were reduced by transfection of P2X4 specific siRNA (37.8±19%, p<0.001) compared to scrambled controls (Fig. 4C).

### The ATP-induced BDNF Expression is Dependent on p38 MAPK Signalling

**Figure 5:**
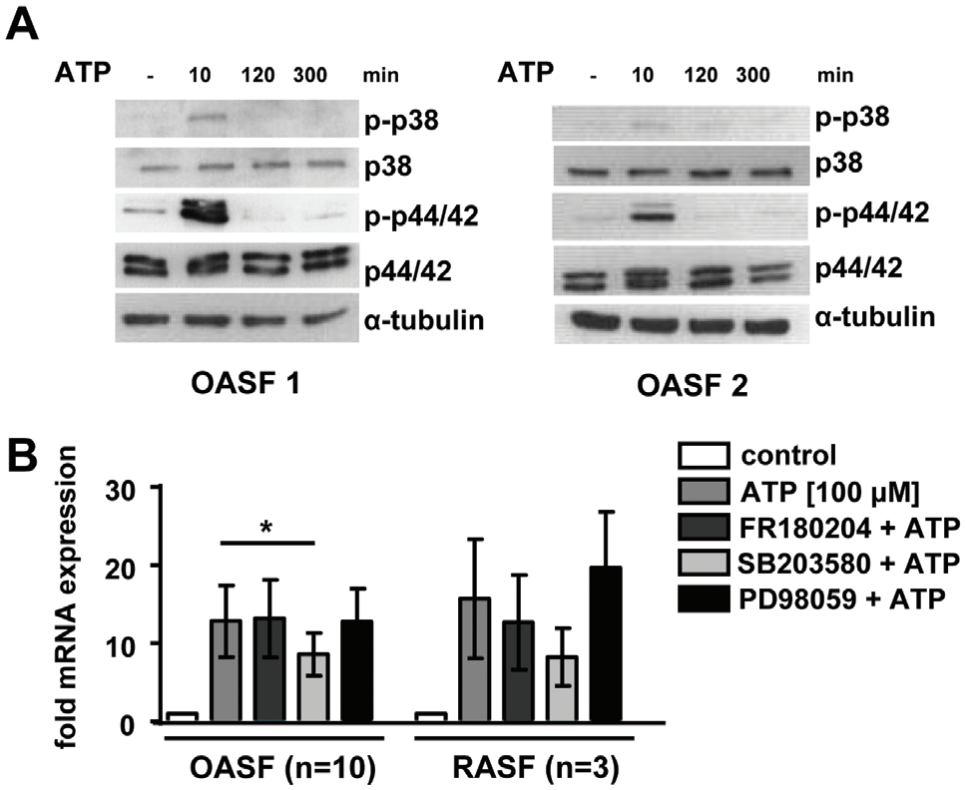
Activation of MAPK pathways in OASF after ATP stimulation. **A**. OASF were stimulated with ATP (100 µM) for 10 minutes, 2 h, 5 h and 24 h. Western blot analysis of p38 and p44/42 MAPK, as well as phosphorylated p-p38 and p-p44/42 MAPK showed kinase activation after 10 minutes ATP stimulation. **B**. OASF (n = 10) and RASF (n = 3) were treated with the kinase inhibitors FR180204, SB203580 and PD98059, respectively 15 min before ATP (100 µM) stimulation for 2 h. Data are shown as means ± standard deviations**.** *, p<0.05.

Since the P2X4 receptor-mediated release of BDNF from rat microglia was shown to be dependent on p38 MAPK signalling [18] we asked, whether this pathway is also active in SF. Therefore, we monitored the phosphorylation of p38 and p44/42 kinases in cell extracts of ATP stimulated OASF in a time course experiment. Both, p38 MAPK and p44/42 MAPK were already phosphorylated within 10 minutes of ATP stimulation (Fig. 5A). The kinase activation was temporally limited since the effect was gone 2 h after starting the ATP treatment. In order to investigate whether these kinases are involved in the ATP-induced expression of BDNF, we treated OASF (n = 10) and RASF (n = 3) with the p38 MAPK inhibitor SB203580, the p44/42 inhibitor FR180204 and the MEK inhibitor PD98059 15 minutes prior ATP stimulation. The ATP-induced mRNA expression of BDNF was significantly reduced in SB203580 pretreated OASF (8.6±2.8 fold, n = 10, p<0.05) compared to controls (12.8±4.6 fold). Pretreatment with other kinase inhibitors had no effect (Fig. 5B). Similar results were obtained in RASF although results did not reach statistical significance due to low patient numbers.

### Transfection of p38 Kinase siRNA Reduced ATP-induced BDNF Expression

**Figure 6:**
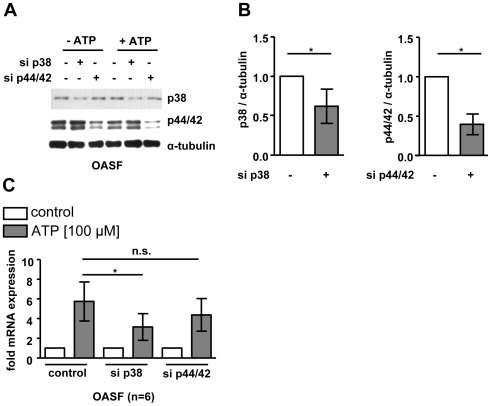
Inhibition of p38 and p44/42 MAPK signaling by siRNA transfection. OASF (n = 6) were transfected with siRNA targeting p38 MAPK, p44/42 MAPK and scrambled siRNA (control) before ATP (100 µM) stimulation for 2 h. **A**. Decreased p38 and p44/42 MAPK expression after siRNA transfection was verified by Western blot analysis. **B**. Densitometric analysis of Western blot results. **C**. The ATP-induced BDNF mRNA expression was reduced in p38 MAPK siRNA transfected OASF but not in p44/42 MAPK siRNA transfected cells. Data are shown as means ± standard deviations**.** *, p<0.05.

To further analyse the involvement p38 and p44/42 MAPK in the hypothesized pathway, we transfected OASF (n = 6) with siRNAs targeting all isoforms of p38 and p44/42 MAPK prior ATP stimulation. The expression levels of p38 (62.0±21.7%, p<0.05) and p44/42 MAPK (39.7±13.1%, p<0.05) were significantly reduced after siRNA transfection compared to controls (Fig. 6A, B). The blockade of p38 (3.2±1.5 fold, p<0.05) but not p44/42 (4.6±1.8 fold, n.s.) MAPK signalling reduced the ATP-induced increase in BDNF mRNA expression compared to scrambled controls (5.7±2.2 fold; Fig. 6C).

## Discussion

For the first time, we provide evidence for the functional expression of the purinergic receptor P2X4 in SF of patients with symptomatic knee OA and RA. Interestingly, P2X4 activation in SF mediates an increase in BDNF expression and release from SF. By using pharmacological, as well as siRNA-mediated inhibition of kinase pathways in SF, we were able to show that the ATP-mediated induction of BDNF is dependent on p38 MAPK signalling but not on other MAPK such as p44/42 or c-Jun aminoterminal kinase (data not shown). Therefore, the underlying basic mechanism in non-glial SF is similar to the signalling cascade recently described for microglia [18]. However, we also observed some differences between BDNF induction in SF and microglia: Trang et al. reported a biphasic release of BDNF at 5 and 60 minutes after ATP stimulation [18], reflecting an immediate release of vesicle-stored BDNF and a delayed secretion of *de novo* synthesized BDNF. We were not able to detect an immediate release of pre-synthesized BDNF within minutes after stimulation as it was shown for microglia. An increased BDNF mRNA synthesis in SF was detectable 2 h after ATP stimulation and the BDNF release was time-delayed with a peak at 5 h. The ATP-induced BDNF expression in rat astrocytes was reported to be mediated by activation of the purinergic receptor P2Y1, Ca^2+^/calmodulin dependent kinase signalling and the phosphorylation of the transcription factor cAMP-responsive element-binding protein (CREB) [22]. Although we observed a phosphorylation of CREB in OASF after 10 minutes ATP stimulation, siRNA experiments reducing endogenous CREB expression levels revealed that CREB is not involved in the ATP-induced transcription of BDNF in SF (see Fig. S1).

Purinergic receptors are expressed in glial cells and P2X/P2Y receptor-mediated signalling critically contributes to the development and maintenance of neuropathic pain [23]. P2X receptor-mediated excitation of nociceptive afferents in normal and arthritic rat knee joints was shown previously [17]. Expression of several P2 purinoreceptors in OA and RA synoviocytes was, at least on the mRNA level, reported previously and a functional role for P2X1, P2X3 and P2X7 in modulation of inflammatory responses was suggested [19], [20]. A couple of purinergic receptors were linked to the development of pain in animal models. The activation of P2X4 in microglia was reported to play a role in tactile allodynia in rats [24]. Moreover, ATP-stimulated BDNF in rat microglia was shown to act as an important signalling molecule between microglia and neurons in pain transmission [25]. Furthermore, disruption of the P2X7 gene in mice abolished chronic inflammatory pain and neuropathic pain [26]. A role for P2Y12 receptors in neuropathic pain development in microglia has been described by two studies in mice [27], [28]. Various P2X purinergic receptor ligands have been patented by diverse pharmaceutical companies in the last years since these compounds may be useful drugs for the treatment of arthritis, pain, inflammation and neurodegenerative diseases [29]. So far, no functional role for purinergic receptors has been described in pain development in human joints. Though, a correlation of synovial fluid ATP levels and OA-associated knee pain in patients has been reported recently [30]. Our double staining of OA synovial tissues revealed that the P2X4 receptor is present in SF but also in other non-fibroblast cell types, most likely endothelial cells and macrophages. The expression of P2X4 in endothelial cells in vessels [31], as well as in macrophages is well established. Recently, the P2X4 mediated release of prostaglandin E2 (PGE_2_) by macrophages was shown to initiate inflammatory pain in mice [32]. Since prostaglandins were also shown to be capable of inducing BDNF secretion from human glial cell lines [33], we excluded an indirect effect of prostaglandins on ATP-induced BDNF expression using a cyclooxygenase inhibitor (see Fig. S2).

Increased BDNF expression levels in the peripheral nerve, dorsal root ganglion (DRG) and dorsal horn have been described in different animal models of neuropathic pain [4]. A local injection of BDNF into the rat hind paw was shown to cause thermal hyperalgesia [34], and exogenous BDNF directly delivered to the intact DRG caused mechanical allodynia [35]. An increased BDNF expression was reported in DRG neurons in the selective spinal nerve ligation animal model [36]. Furthermore, BDNF has been described in inflammatory pain states. In humans, BDNF expression in the pancreas is upregulated and associated with pain in chronic pancreatitis [7]. In rats, BDNF expression levels in bladder were increased in models of bladder inflammation [6]. Moreover, colonic BDNF expression levels were increased in mouse models of inflammatory bowel disease, suggesting a role for BDNF in colonic hypersensitivity [5], [8]. The expression of BDNF in human SF was reported earlier [13], [16] and BDNF was shown to be particularly expressed under more inflammatory conditions in synovial tissue [16]. Our results also showed an increased BDNF secretion from RASF compared to OASF in unstimulated conditions. Barthel et al. also reported a slightly elevated BDNF mRNA expression in synovial fluid cells of RA compared to OA patients [12]. However, it has to be noted that our results in OASF indicate that basal BDNF levels highly vary amongst patients and it seems that central acting medication strongly increases BDNF expression levels. The OA patients exhibiting 14-fold increased basal BDNF levels were medicated with amitriptyline, the opioid analgesic oxycodon and the benzodiazepine oxazepam. Increased serum BDNF concentrations were reported in depressed patients treated with amitriptyline [37] and this drug was shown to induce the production of BDNF in whole blood cell cultures derived from healthy individuals [38]. To our knowledge, no literature linking oxycodon and oxazepam, respectively with BDNF is available. However, other benzodiazepines [39], [40] as well as other opioides [41], [42], [43] were shown to increase BDNF expression levels.

The fact that SF are capable of secreting BDNF actively upon increased ATP levels, a signal of inflamed and damaged tissue, is entirely new. So far, there are few data available about other signals than ATP regulating BDNF secretion in diverse cell types. An increased BDNF secretion has been observed upon stimulation of monocytes, B- and T- cells [44]. Cytokines, such as tumor necrosis factor- α (TNF- α) and interleukin-6 (IL-6) were shown to induce BDNF secretion from monocytes [45]. Stimulation of microglia cells with IL-17 resulted in increased BDNF, nerve growth factor (NGF) and glia-derived neurotrophic factor (GDNF) expression levels and release [46]. Raychaudhuri et al. showed that stimulation of SF with IL1- β and TNF- α resulted in an increased NGF release, but BDNF expression and release were not studied [47].

In conclusion, we showed that the ATP-induced expression and release of the neurotrophin BDNF is dependent on P2X4 and on p38 MAPK signalling. It is interesting that the pathway operating in microglia is also observed in non-glial SF. A potential functional role for BDNF in pain transmission in synovial tissues *in vivo* remains to be investigated.

## Materials and Methods

### Tissue Samples and Cell Culture

SF were obtained from symptomatic knee OA and RA patients that underwent joint replacement surgery in the Schulthess Clinic Zurich. Informed consent was obtained from all patients. All patients fulfilled the criteria for the classification of OA [48] and RA [49], respectively. Cells were cultured as described elsewhere [50] and used between passages 4 to 9 for all experiments.

### Stimulation of Cells

For stimulation experiments 2×10^5^ cells were cultured in 6-well plates. 24 h prior stimulation, cells were serum starved in DMEM/F-12 (Invitrogen, Basel, Switzerland) supplemented with 0.5% fetal calf serum (FCS; Invitrogen). SF were stimulated with ATP (50, 100, 200, 500 µM; Sigma-Aldrich, Buchs, Switzerland) for 10 min, 2 h, 5 h and 24 h, as well as ADP (100 µM; Sigma-Aldrich) and UTP (100 µM; Sigma-Aldrich) for 2 h. In kinase inhibitor experiments, cells were treated with 5 µg/ml of the p38 MAPK inhibitor SB203580 (Calbiochem, Darmstadt, Germany), 30 µM of the ERK inhibitor II FR180204 (Calbiochem) and 50 µM of the MAPK kinase (MEK) inhibitor PD98059 (Calbiochem) 15 minutes prior ATP stimulation for 2 h. Cells were harvested at indicated time points for isolation of RNA and Western blot analysis, respectively. Cell culture supernatants were collected and stored at −80°C for enzyme-linked immunosorbent assay (ELISA) experiments.

### Isolation of RNA, Reverse Transcription, and Quantitative Real-Time PCR

Total RNA was isolated using the RNeasy Mini Kit (Qiagen, Hombrechtikon, Switzerland) including on-column DNaseI (Qiagen) digest and reversed transcribed using MultiScribe reverse transcriptase and random hexamer primers (Applied Biosystems, Rotkreuz, Switzerland). Real-time PCR was performed on the ABI PRISM 7700 Sequence Detector System (Applied Biosystems) by using the TaqMan Gene Expression Assays Hs00380947_m1 and Hs00602442_m1 for human BDNF and P2X4 (Applied Biosystems), respectively. Constitutively expressed human glyceraldehyde 3-phosphate dehydrogenase (GAPDH) was measured for internal standard sample normalization using SYBR Green (Applied Biosystems) and primers as described elsewhere [51]. Relative mRNA expression levels were calculated by the comparative threshold cycle method (ΔΔCt).

### BDNF ELISA

Cell culture supernatants from unstimulated as well as from ATP stimulated cells were concentrated using Amicon Ultra 0.5 ml Centrifugal Filters (Milian AG, Geneva, Switzerland) with a molecular weight cut off of 10 kDa. BDNF release into cell culture supernatants was measured using the RayBio Human BDNF ELISA kit (Lucerna-Chem AG, Luzern, Switzerland) according to the manufacturer’s instructions.

### Western Blot Analysis

Cells from 6-well plates were lysed directly in 200 µl Lemmli buffer (62.5 mM TrisHCl, 2% SDS, 10% Glycerol, 0.1% Bromphenolblue, 5 mM β-mercaptoethanol). Whole cell lysates were separated on 10% SDS polyacrylamide gels and electroblotted onto nitrocellulose membranes (Whatman, Dassel, Germany). Membranes were blocked for 1 h in 5% (w/v) non-fat milk in TBS-T (20 mM Tris base, 137 mM sodium chloride, 0.1% Tween 20, pH 7.6). After blocking, the membranes were probed with antibodies against P2X4 receptor (Alomone Labs, Jerusalem, Israel; 1∶200), p38 MAPK (Cell Signaling, Allschwil, Switzerland; 1∶1000), phospho-p38 (p-p38) MAPK (Cell Signaling; 1∶1000), p44/42 MAPK (Cell Signaling; 1∶1000), phospho-p44/42 (p-p44/42) MAPK (Cell Signaling; 1∶1000), and α-tubulin (Abcam, Cambridge, UK; 1∶5000), respectively. After three washes with TBS-T, the horseradish peroxidase-conjugated secondary goat anti-rabbit antibody or goat anti-mouse antibody (Jackson ImmunoResearch, Suffolk, UK) was added at a dilution of 1∶5000 in 5% (w/v) non-fat milk/TBS-T for 1 h. Blots were washed three times in TBS-T, followed by detection with the ECL Western blotting detection reagents (GE Healthcare, Glattbrugg, Switzerland) and exposured on Hyperfilm ECL (GE Healthcare). Evaluation of the expression of specific proteins was performed by the Alpha Imager Software system (Alpha Innotech, San Leandro, CA, USA) via pixel quantification of the electronic image.

### Transfection of Cells with siRNA

2×10^5^ cells were cultured one day before transfection in 6-well plates. Per transfection (P2X4), 125 µl serum-free OptiMEM (Invitrogen, Basel, Switzerland) were mixed with 5 µl Lipofectamine 2000 reagent (Invitrogen). After 5 minutes, 125 µl serum-free OptiMEM containing either siRNA (Qiagen) targeting P2X4 (Hs_P2RX4_5) or scrambled siRNA (Ctrl_AllStars_1) were added. The transfection solutions were incubated for 20 minutes at room temperature and then added to 1000 µl of DMEM/F-12 supplemented with 10% FCS without antibiotics contained in each well. The final concentration of siRNA per well was 40 nM. After 72 h, cells were retransfected for additional 48 h using the same protocol. 24 h after the second transfection, cells were serum starved in DMEM/F-12 supplemented with 0.5% FCS and treated the day after with 100 µM ATP for 2 h. For the transfection of siRNAs targeting p38 MAPK, p44/42 MAPK or scrambled SignalSilence Control siRNA (Cell Signaling, Allschwil, Switzerland) the Amaxa Basic Nucleofector Kit for Primary Mammalian Fibroblasts (Lonza, Basel, Switzerland) was used. 2×10^5^ cells per condition (± ATP) were resuspended in 100 µl of Nucleofector Solution and 200 nM siRNA were added. The transfection was carried out in a cuvette using the program U-23 of the Amaxa Nucleofector device. Afterwards, 500 µl of DMEM/F-12 supplemented with 10% FCS were added immediately. The suspension of one cuvette was divided into 2 wells on a 6-well plate. 48 h after transfection, cells were serum starved in DMEM/F-12 supplemented with 0.5% FCS and treated the day after with 100 µM ATP for 2 h. Cells were harvested, and total cellular RNA and whole-cell lysates were extracted.

### Immunohistochemistry

After deparaffinization, sections were pre-treated with citrate buffer (10 mM sodium citrate, pH 6.0). Endogenous peroxidase activity was disrupted with 3% H_2_O_2_. Nonspecific protein binding was blocked with 1% bovine serum albumin (BSA)/5% goat serum for 40 minutes. Polyclonal rabbit anti-P2X4 antibodies (Alomone Labs) and rabbit IgG1 (isotype control) were applied (8 µg/ml) over night at 4°C. Slides were washed in PBS-T (0.05% Tween 20 in PBS) and incubated with biotinylated goat anti-rabbit antibodies (1∶1000; Jackson ImmunoResearch). The signal was amplified with ABC reagent (Vector Laboratories, Burlingame, CA) and detected with 3,3̀-diaminobenzidine (DAB; Vector Laboratories). The next day, slides were blocked as described above and incubated with monoclonal mouse anti-prolyl-4-hydroxylase-β (Acris Antibodies, Hiddenhausen, Germany), a marker enzyme for fibroblasts and as an isotype control, mouse IgG1 (10 µg/ml). Slides were washed and incubated with biotinylated goat anti-mouse antibodies (1∶2000; Jackson ImmunoResearch). The signal was amplified using ABC reagent and detected with HistoGreen (Linaris, Wertheim-Bettingen, Germany) as a substrate.

### Statistical Analysis

Statistical analysis on data sets was carried out by using the GraphPad Prism program (GraphPad Software, San Diego, CA). Differences between experimental groups were analysed by one-way analysis of variance (ANOVA) with Tukey’s post hoc test. Differences in basal BDNF levels between OASF and RASF were analysed by unpaired t test. Wilcoxon signed-rank test was used to analyse the differences of densitometric intensities of Western blot results in siRNA experiments. Data are reported as means ± standard deviations. P values <0.05 were considered significant.

## Supporting Information

Figure S1 **cAMP-responsive element-binding protein (CREB) is not involved in the ATP-induced regulation of BDNF expression.** **A**. OASF were stimulated with ATP (100 µM) for 10 minutes, 2 h, 5 h and 24 h. Western blot analysis of p38 and p44/42 MAPK, phosphorylated p-p38 and p-p44/42 MAPK, as well as phosphorylated p-CREB showed kinase and CREB activation after 10 minutes ATP stimulation. **B**. The reduction of endogenous CREB levels by siRNA transfection in OASF (n = 3) did not affect the **C**. ATP-mediated increase in BDNF mRNA expression. Data are shown as means ± standard deviations. N.s., not significant; **, p<0.01.
(TIF)

Figure S2 **Cyclooxygenase (COX) is not involved in the** **ATP-induced regulation of BDNF expression.** OASF (n = 6) were stimulated with the COX inhibitor sc-560 (100 nM) and ATP (100 µM) for 2 h. The ATP-mediated increase in BDNF expression was not changed by COX-inhibitor treatment. Data are shown as means ± standard deviations. N.s., not significant; *, p<0.05, **, p<0.01.
(TIF)
