# Supplementary figures and images for: ATP Induced Brain-Derived Neurotrophic Factor Expression and Release from Osteoarthritis Synovial Fibroblasts Is Mediated by Purinergic Receptor P2X4
Source: PLoS One. 2012 May 25;7(5):e36693. doi: 10.1371/journal.pone.0036693 (PMC3360754; doi:10.1371/journal.pone.0036693)

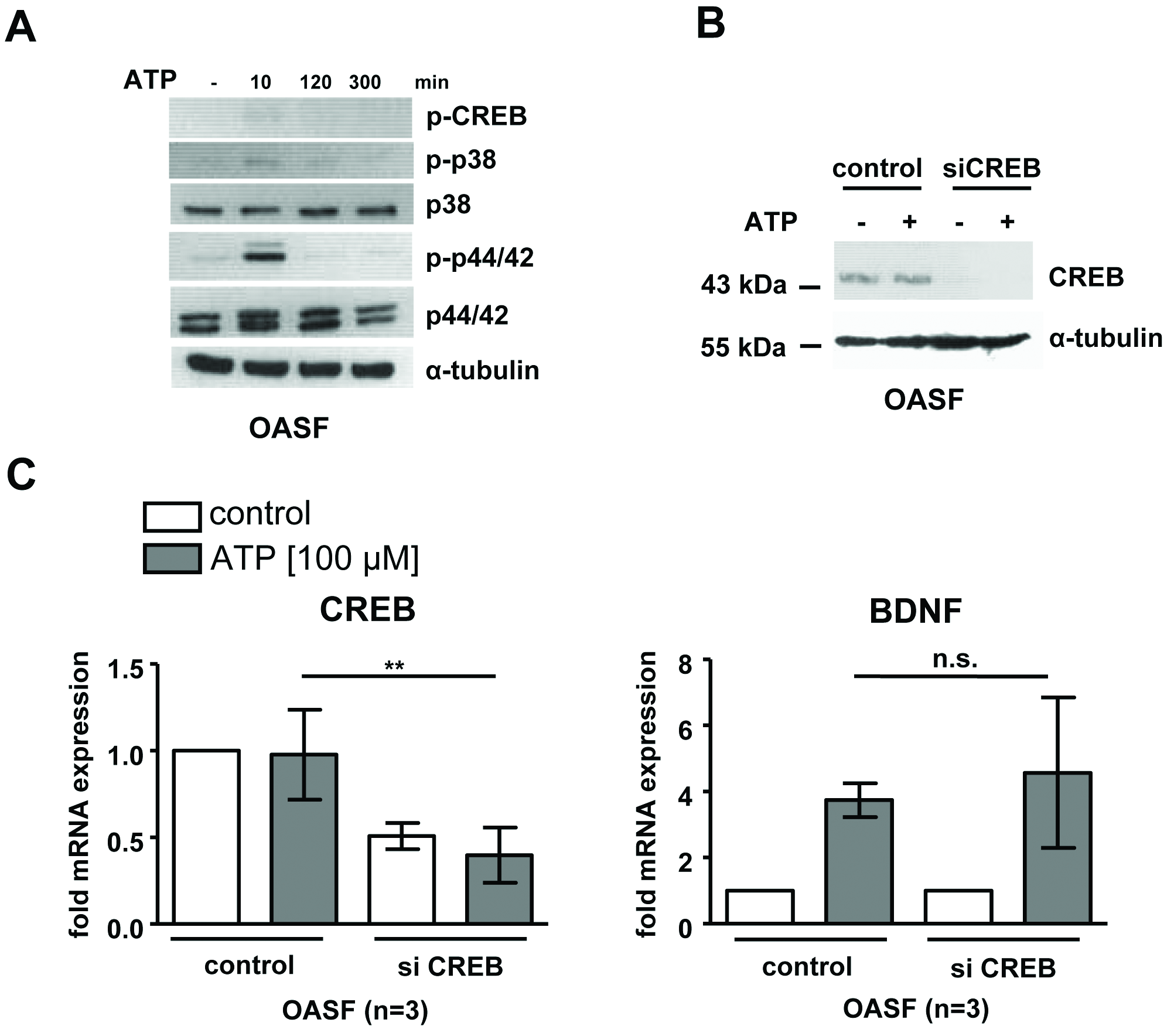

Supplement: Figure S1 — cAMP-responsive element-binding protein (CREB) is not involved in the ATP-induced regulation of BDNF expression. A. OASF were stimulated with ATP (100 µM) for 10 minutes, 2 h, 5 h and 24 h. Western blot analysis of p38 and p44/42 MAPK, phosphorylated p-p38 and p-p44/42 MAPK, as well as phosphorylated p-CREB showed kinase and CREB activation after 10 minutes ATP stimulation. B. The reduction of endogenous CREB levels by siRNA transfection in OASF (n = 3) did not affect the C. ATP-mediated increase in BDNF mRNA expression. Data are shown as means ± standard deviations. N.s., not significant; **, p<0.01. (TIF) [file pone.0036693.s001.tif]

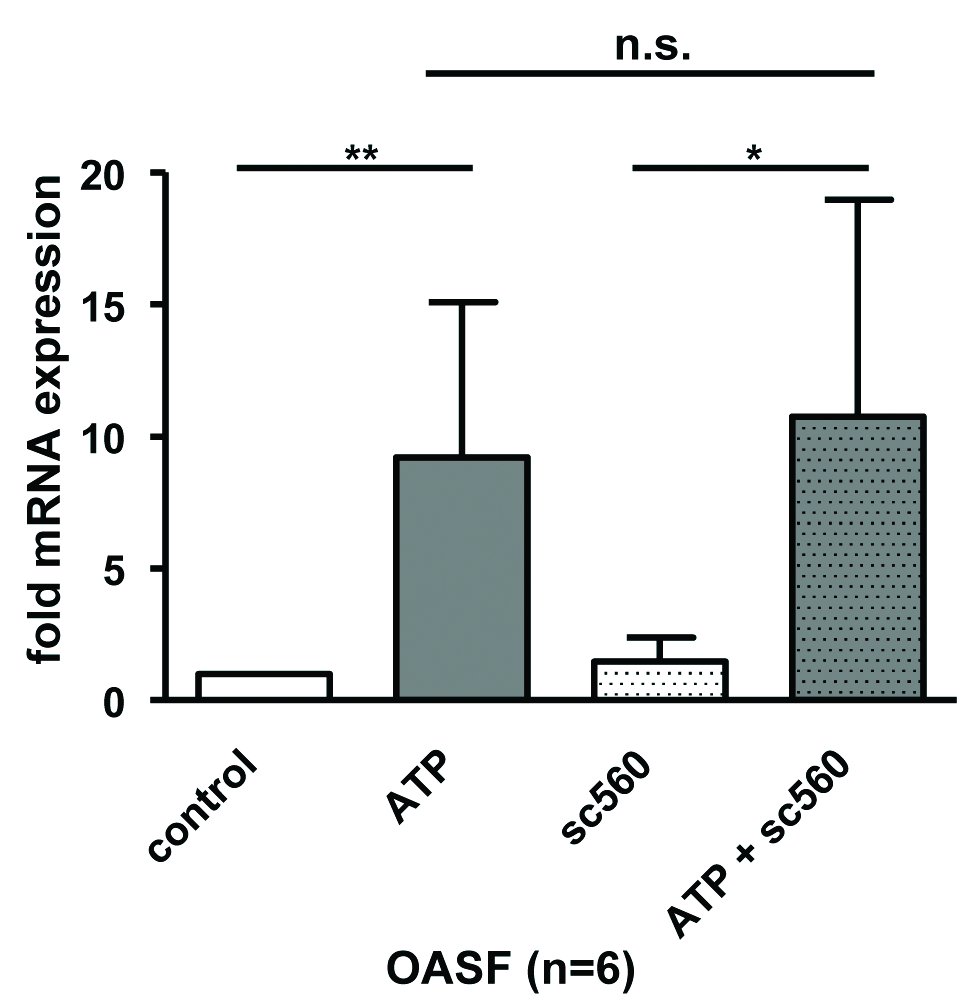

Supplement: Figure S2 — Cyclooxygenase (COX) is not involved in the ATP-induced regulation of BDNF expression. OASF (n = 6) were stimulated with the COX inhibitor sc-560 (100 nM) and ATP (100 µM) for 2 h. The ATP-mediated increase in BDNF expression was not changed by COX-inhibitor treatment. Data are shown as means ± standard deviations. N.s., not significant; *, p<0.05, **, p<0.01. (TIF) [file pone.0036693.s002.tif]
